# Deep learning-based automated segmentation of intracerebral haemorrhage, intraventricular haemorrhage and perihaematomal oedema on non-contrast CT

**DOI:** 10.1093/esj/aakag007

**Published:** 2026-03-07

**Authors:** Floor N H Wilting, Jules P J Douwes, Ajay Patel, Floris H B M Schreuder, Ruben Dammers, Gerjon Hannink, Wilmar M T Jolink, Sjoert A H Pegge, Lotte Sondag, Marieke J H Wermer, H Bart van der Worp, Frederick J A Meijer, Catharina J M Klijn

**Affiliations:** Department of Neurology, Donders Institute for Brain, Cognition and Behaviour, Radboud University Medical Center, Nijmegen, The Netherlands; Department of Neurology and Neurosurgery, Brain Center, University Medical Center Utrecht, Utrecht, The Netherlands; Department of Otorhinolaryngology-Head and Neck Surgery, Leiden University Medical Center, Leiden, The Netherlands; Department of Medical Imaging, Radboud University Medical Center, Nijmegen, The Netherlands; Department of Neurology, Donders Institute for Brain, Cognition and Behaviour, Radboud University Medical Center, Nijmegen, The Netherlands; Department of Neurosurgery, Erasmus University Medical Center, Erasmus MC Stroke Center, Center for Complex Microvascular Surgery, Rotterdam, The Netherlands; Department of Medical Imaging, Radboud University Medical Center, Nijmegen, The Netherlands; Department of Neurology, Isala Hospital, Zwolle, The Netherlands; Department of Medical Imaging, Radboud University Medical Center, Nijmegen, The Netherlands; Department of Neurology, Donders Institute for Brain, Cognition and Behaviour, Radboud University Medical Center, Nijmegen, The Netherlands; Department of Neurology, Jeroen Bosch Hospital, ‘s-Hertogenbosch, The Netherlands; Department of Neurology, University Medical Center Groningen, Groningen, The Netherlands; Department of Neurology, Leiden University Medical Center, Leiden, The Netherlands; Department of Neurology and Neurosurgery, Brain Center, University Medical Center Utrecht, Utrecht, The Netherlands; Department of Medical Imaging, Radboud University Medical Center, Nijmegen, The Netherlands; Department of Neurology, Donders Institute for Brain, Cognition and Behaviour, Radboud University Medical Center, Nijmegen, The Netherlands

**Keywords:** deep learning, intracerebral haemorrhage, intraventricular haemorrhage, perihaematomal oedema, automated segmentation, non-contrast computed tomography, artificial intelligence

## Abstract

**Introduction:**

Precise volumetric evaluation of intracerebral haemorrhage (ICH), intraventricular haemorrhage (IVH) and perihaematomal oedema (PHO) is essential but manual segmentation is time-consuming and susceptible to variability. We aimed to develop and externally validate a deep learning model for simultaneous segmentation of ICH, IVH and PHO on non-contrast CT (NCCT) in patients with spontaneous ICH.

**Patients and methods:**

A 3D U-net model was trained with 5-fold cross-validation on baseline NCCTs from 301 patients included in 2 prospective multicentre studies. External validation was performed on 141 baseline NCCTs from another multicentre study. Model performance was evaluated against manual ground truth segmentations using the Dice similarity coefficient (DSC), intraclass correlation coefficients (ICC) and Bland–Altman analyses.

**Results:**

The model achieved a median DSC of 0.93 (IQR 0.91–0.94) for ICH, 0.75 (IQR 0.57–0.82) for IVH and 0.53 (IQR 0.34–0.65) for PHO. Volume correlations were excellent for ICH (mean absolute and consistency ICC both 0.98 [95% CI 0.98–0.99]) and IVH (absolute ICC 0.97 [95% CI 0.92–0.98]; consistency ICC 0.98 [95% CI 0.96–0.99]), and moderate for PHO (absolute ICC 0.60 [95% CI -0.08–0.85]; consistency ICC 0.82 [95% CI 0.76–0.87]). Bland–Altman analyses demonstrated a bias for ICH of −0.48 mL (LoA −8.21 to 7.26), for IVH of −1.68 mL (LoA −7.35 to 3.99) and for PHO of 13.91 mL (LoA −4.85 to 32.68).

**Discussion and conclusion:**

The model enables accurate automated segmentation of ICH, while IVH and PHO segmentation remain more challenging. Automated segmentations may already serve as reliable pre-segmentations in research, but require visual assessment and correction, in particular for IVH and PHO.

## Graphical Abstract

**Graphical Abstract:**
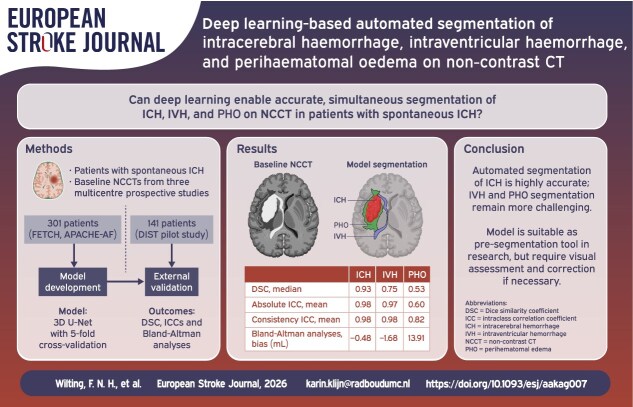

## Introduction

Intracerebral haemorrhage (ICH) is a subtype of stroke associated with high morbidity and mortality.^1^ ICH causes direct injury by physical disruption of the brain parenchyma and exertion of mass effect.^2^ In up to 50%, the haematoma extends into the ventricular system, resulting in intraventricular haemorrhage (IVH) and potentially hydrocephalus due to disrupted cerebrospinal fluid flow.^3^^,^^4^ Additionally, the intraparenchymal blood induces an inflammatory response, which, together with the release of neurotoxic clot components, results in secondary brain injury.^2^^,^^5^ The formation of perihaematomal oedema (PHO) is a key manifestation of secondary brain injury and may further aggravate the deleterious mass effect of the haematoma.

Volumes of ICH, IVH and PHO are independent predictors of functional outcome after ICH, with poorer outcomes when volumes are larger.^6–8^ Therefore, they are key variables to characterise patient populations and to ensure comparability across observational studies and randomised clinical trials. Moreover, ICH volume is frequently used as a trial eligibility criterion, while PHO is increasingly recognised as a surrogate marker for secondary brain injury after ICH.^9^

For volumetric assessment, semi-quantitative methods such as the ABC/2 for ICH^10^ and the modified Graeb Score (mGS) for IVH^11^ are commonly used, as they enable rapid approximation of ICH and IVH volumes. However, these methods have limitations. The ABC/2 method tends to underestimate volumes of irregularly-shaped and larger haemorrhages,^12^^,^^13^ while the mGS provides an ordinal score rather than a volumetric assessment in millilitres. Furthermore, no rapid method exists for quantifying PHO. Manual segmentation by expert radiologists is therefore still considered the gold standard, but is laborious, costly and prone to interrater variability.

To overcome the drawbacks of manual segmentation, deep learning-based models have been introduced to automatically segment medical images.^14^ Recent advances in artificial intelligence have markedly improved their performance and efficiency, making them increasingly popular and indispensable in the field of medical image analysis, including neuroradiology.^15^

Over the past decade, various segmentation models have been developed for non-traumatic ICH.^16^ However, many focus on intracerebral blood without simultaneous segmentation of IVH,^17^^,^^18^ PHO,^19–29^ or both.^30–37^ Furthermore, these studies did not distinguish between intraparenchymal and intraventricular haemorrhage,^19–23^^,^^25–27^^,^^29^^,^^38^ lacked external validation^17^^,^^19^^,^^26–28^^,^^30–37^^,^^39^ or were limited to single-centre data.^19^^,^^22^^,^^23^^,^^26^^,^^27^^,^^30–32^^,^^35–37^^,^^40^ Therefore, the aim of this study was to develop and externally validate a deep learning-based model for simultaneous segmentation of ICH, IVH and PHO on multicentre non-contrast brain CT (NCCT) in patients with spontaneous ICH.

## Patients and methods

### Study population

**Figure 1:**
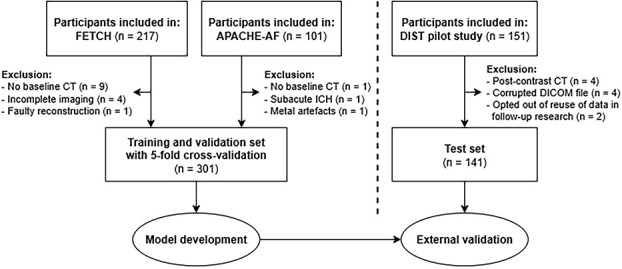
Flow chart of participants included in the training/validation and test sets.

We retrospectively included NCCTs from patients with a spontaneous ICH who participated in three prospective studies conducted in the Netherlands (Figure 1).^41–43^ All studies were approved by the institutional review board, and written informed consent was obtained from all study participants or their legal representatives in accordance with the declaration of Helsinki. Participants could object to use of their data in follow-up research, in which case they were excluded from the current project.

Baseline NCCTs of 301 participants from the FETCH (Finding the Etiology in spontaneous Cerebral Hemorrhage; *n* = 203 participants) and APACHE-AF (APixaban versus Antiplatelet drugs or no antithrombotic drugs after anticoagulation-associated ICH in patients with Atrial Fibrillation; *n* = 98 participants) studies were used for model development. FETCH was a multicentre (3 sites), prospective observational cohort study enrolling patients with a spontaneous ICH between 1 October 2013 and 31 December 2018.^41^ APACHE-AF was a multicentre (16 sites), randomised clinical trial enrolling patients with atrial fibrillation who experienced a spontaneous ICH during anticoagulation therapy between 15 January 2015 and 6 July 2020, comparing anticoagulant reinitiation versus avoidance.^42^ For external validation, baseline NCCTs of 141 participants from the Dutch ICH Surgery Trial (DIST) pilot study were used. The DIST pilot study was a multicentre (10 sites), prospective non-randomised intervention study investigating minimally invasive endoscopy-guided surgery within 8 h of symptom onset for spontaneous supratentorial ICH between 27 November 2018 and 31 October 2021.^43^

Inclusion and exclusion criteria of these studies have been described previously.^41–43^ Baseline NCCTs were acquired as part of routine clinical care at hospital presentation, using scanners from multiple vendors (GE HealthCare, Philips, Siemens and Toshiba/Canon Medical) with a mix of helical and volume acquisition techniques. Most protocols used a tube voltage between 100 and 120 kV and a tube current between 100 and 400 mA. Images were reconstructed using the vendor’s default brain kernel. Both thin-slice (≤1 mm) and thick-slice (3–5 mm) axial images were included for model development and external validation.

### Reference standard

Manual voxel-wise segmentations of ICH, IVH and PHO on baseline NCCTs from FETCH and APACHE-AF were obtained to train the model. To further improve differentiation between intraparenchymal, intraventricular and extra-axial blood, additional segmentations of subarachnoid haemorrhage (SAH) and subdural haemorrhage (SDH) were included. Segmentations were performed using ITK-SNAP (version 3.8.0, https://www.itksnap.org/) with window width/level settings of 80/40 Hounsfield units (HU). A single trained rater (J.P.J.D.) with 2 years of research experience in ICH performed the segmentations. Prior to study initiation, the rater received dedicated training in brain CT interpretation and segmentation from a vascular neurologist (F.H.B.M.S.) and vascular neuroradiologist (F.J.A.M.), both with over 10 years of experience. All segmentations were subsequently reviewed and verified by the same vascular neurologist and neuroradiologist.

For the DIST pilot study, ground truth segmentations of ICH, IVH and PHO were available from prior manual segmentation (S.A.H.P.; vascular neuroradiologist with over 10 years of experience). As only ICH, IVH and PHO segmentations were available in the DIST pilot study dataset, and because presence of SAH and SDH was relatively rare, external validation was restricted to these 3 labels.

### Deep learning model

To develop a deep learning-based model for simultaneous ICH, IVH and PHO segmentation, a 3D U-Net model was trained using the nnU-Net framework with default configurations (https://www.github.com/MIC-DKFZ/nnUNet/tree/nnunetv1).^44^ The self-configuring nnU-Net automatically determines what is required for data preprocessing, network architecture, training and postprocessing. All NCCTs were resampled to a uniform voxel spacing of 0.6 × 0.43 × 0.43 mm prior to model training, corresponding to the median voxel spacing of the training data. Training was performed on an NVIDIA TITAN X GPU with 5-fold cross-validation for 1000 epochs, each with 250 mini-batches with a batch size of 2 and patch dimensions of 96 × 160 × 160 voxels. The final 3D nnU-Net architecture comprised approximately 25 million trainable parameters. The detailed model architecture is shown in Figure S1. At inference, the five models resulting from the 5-fold cross-validation were used as an ensemble to produce the final test case segmentations.

### Statistical analyses and model performance evaluation

Categorical data were reported as absolute numbers ($n$) with relative percentages and compared using the ${\chi}^2$ test or Fisher’s exact test, as appropriate. Continuous data were summarised as medians with interquartile ranges (IQR) and compared using the Mann–Whitney U test.

Model performance was evaluated by comparing test set segmentations generated by the ensembled model with manual ground truth segmentations. The primary performance metric was the Dice similarity coefficient (DSC), which quantifies spatial overlap between two segmentations on a scale from 0 (no overlap) to 1 (perfect overlap).^45^ Reliability and agreement between model-predicted and manual segmentations were further assessed using absolute and consistency intraclass correlation coefficients (ICCs) and Bland–Altman analyses,^46^ and were reported with 95% CI. Subgroup analyses of DSC scores were performed to explore differences in model performance according to ICH location (deep vs. lobar) and presence of IVH. Detailed definitions of all performance metrics are provided in the Supplementary material. In addition to quantitative evaluation, model-predicted segmentations were visually assessed to identify common patterns of misclassification and boundary ambiguity.

To identify outliers that could distort ICC and Bland–Altman analyses, linear regression models of model-predicted versus manually segmented volumes were fitted for each segmentation label. Influence diagnostics were performed using DFBETA values, which quantify the change in the estimated regression coefficient when a specific data point is omitted (Supplementary material). Data points were considered influential outliers if the DFBETA for the intercept exceeded the threshold of $\pm 2/\surd n$, where $n$ is the sample size.^47^ The ICC and Bland–Altman analyses were conducted with and without the identified outliers. For the primary analyses, the analyses without the outliers were used.

All data analyses were performed using Python (version 3.9; https://www.python.org) and R (version 4.4.1; R Foundation for Statistical Computing). A *P* < .05 was considered statistically significant.

## Results

### Study population

Baseline characteristics of the training/validation and test sets are presented in Table 1. Participants in the test set were younger (median age 66 vs. 72 years, *P* = .008) and more often had deep ICH (54.6% vs. 43.9%, *P* = .04), while infratentorial ICH was present exclusively in the training/validation set. Median ICH and IVH volumes were larger in the test set (ICH: 27.0 mL; IVH: 9.7 mL) compared to the training/validation set (ICH: 9.0 mL; IVH 3.1 mL, both *P* < .001). PHO volumes in the training/validation and test sets were similar.

**Table 1:** Baseline characteristics of participants included in the training/validation and test sets.

Characteristic	Training/Validation Set ($\boldsymbol{n}$ = 301)	Test Set ($\boldsymbol{n}$ = 141)	*P*-value
**Age, years; median [IQR]**	72 [61–80]	66 [58–76]	.008
**Sex;** $\boldsymbol{n}$ **(%)**			.98
**Male**	192 (63.8)	89 (63.1)	
**Female**	109 (36.2)	52 (36.9)	
**ICH location;** $\boldsymbol{n}$ **(%)**	^a^		<.001
**Lobar**	113 (37.5)	64 (45.4)	.14
**Deep**	132 (43.9)	77 (54.6)	.04
**Infratentorial**	55 (18.3)^b^	0 (0.0)	<.001
**Brainstem**	12 (4.0)		
**Cerebellum**	47 (15.6)		
**Intraventricular extension;** $\boldsymbol{n}$ **(%)**	121 (40.2)	69 (48.9)	.10
**Subarachnoid extension;** $\boldsymbol{n}$ **(%)**	81 (26.9)	42 (29.8)	.61
**Subdural extension;** $\boldsymbol{n}$ **(%)**	9 (3.0)	3 (2.1)	.76
**ICH volume, mL; median [IQR]**	9.0 [3.8–22.4]	27.0 [15.7–47.9]	<.001
**IVH volume, mL; median [IQR]**	3.1 [1.2–12.4]	9.7 [3.9–20.9]	<.001
**PHO volume, mL; median [IQR]** ^c^	11.0 [6.0–23.1]	12.5 [5.6–23.8]	.95

Abbreviations: ICH = intracerebral haemorrhage; IQR = interquartile range; IVH = intraventricular haemorrhage; *n* = number; PHO = perihaematomal oedema.

a One patient had a primary intraventricular haemorrhage without intraparenchymal involvement.

b Four patients had both brainstem and cerebellar involvement.

c PHO was present in 299/301 patients in the training/validation set, and 137/141 patients in the test set.

### Model-predicted versus manual segmentations

**Figure 2:**
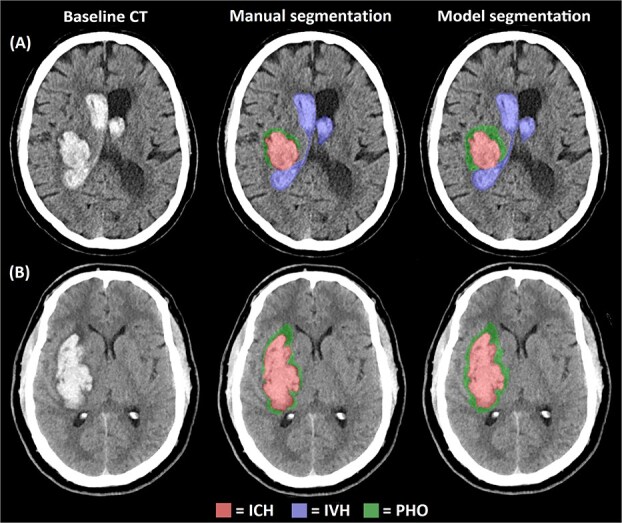
Illustrative examples demonstrating the baseline non-contrast CTs and corresponding manual and model-predicted segmentations of intracerebral haemorrhage (ICH), intraventricular haemorrhage (IVH) and perihaematomal oedema (PHO). (A) Patient with a deep haemorrhage with IVH and PHO. (B) Patient with a deep haemorrhage with PHO and no IVH.

Illustrative examples of model-predicted and manual segmentations are shown in Figure 2. ICH was present in all participants and consistently detected by the model, with comparable segmented volumes (model: median 28.9 mL [IQR 15.5–45.9]; manual: 27.0 mL [IQR 15.7–47.9]). For IVH, the model correctly identified intraventricular blood in 68 of 69 participants, with slightly lower median volumes than manual segmentations (7.8 mL [IQR 2.7–17.5] vs. 9.7 mL [IQR 4.0–21.2]). However, false-positive IVH segmentations were observed in 34 participants (24.1%) without rater-confirmed IVH, generally with very small volumes (median 0.06 mL [IQR 0.03–0.25]). Conversely, IVH was missed in one participant (0.7%) in whom a small amount of IVH was manually segmented (0.41 mL). For PHO, the model segmented oedema in 137 of 141 participants (97.2%), with median volumes substantially larger than manual segmentations (31.4 mL [IQR 18.9–43.7] vs. 12.5 mL [IQR 5.6–23.8]). In addition, PHO was segmented in four participants (2.8%) in whom the manual rater did not identify oedema, with median volumes of 17.2 mL (IQR 16.2–20.4).

**Figure 3:**
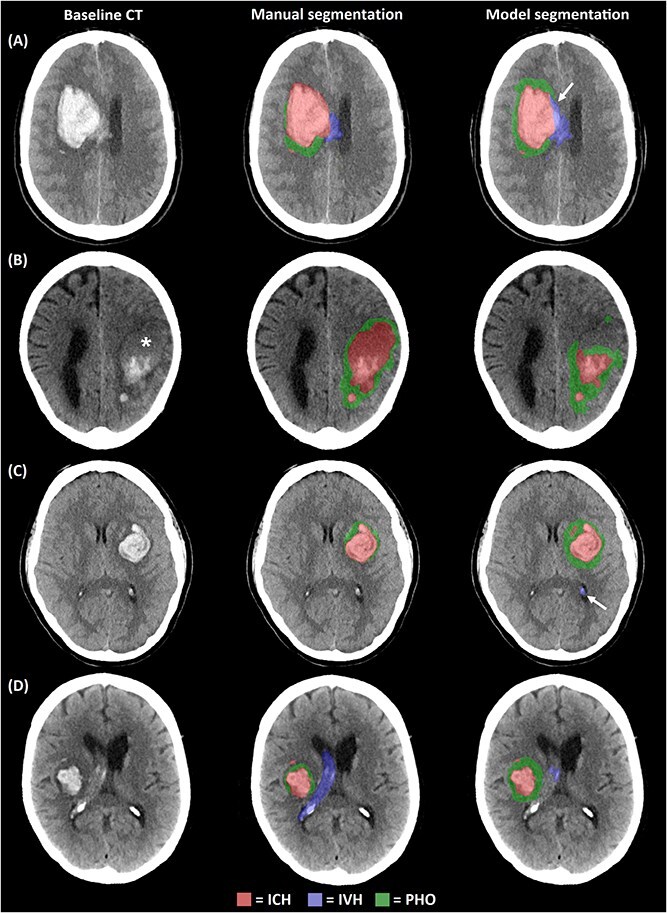
Examples of segmentation errors by the model. (A) Misclassification of intracerebral haemorrhage (ICH) at the ventricular border as intraventricular haemorrhage (IVH) (arrow) and relative perihaematomal oedema (PHO) oversegmentation. (B) Blend sign (asterisk) not recognised as ICH, resulting in ICH undersegmentation. (C) False-positive segmentation of hyperdense choroid plexus as IVH (arrow) and relative PHO oversegmentation. (D) Undersegmentation of less dense IVH and relative PHO oversegmentation.

Visual assessment provided further insight into model-predicted segmentations. For ICH, misclassification occasionally occurred at the ventricular border, where blood was segmented as IVH (Figure 3A), or vice versa. Moreover, hypo- or isodense regions within the haematoma (eg, blend signs or fluid levels)^48^ were sometimes not recognised as ICH (Figure 3B). For IVH, false-positive segmentations most often corresponded to hyperdense choroid plexus (Figure 3C), small haemorrhages separate from the main haematoma (satellite signs) or parenchymal blood adjacent to the ventricles, while undersegmentation occurred when intraventricular blood was less dense (Figure 3D). For PHO, the model generally produced larger segmentations compared with the manual segmentations, particularly in deep ICH where differentiation from white matter or white matter lesions can be ambiguous (Figure 3A,C and D).

### Overall model performance

**Figure 4:**
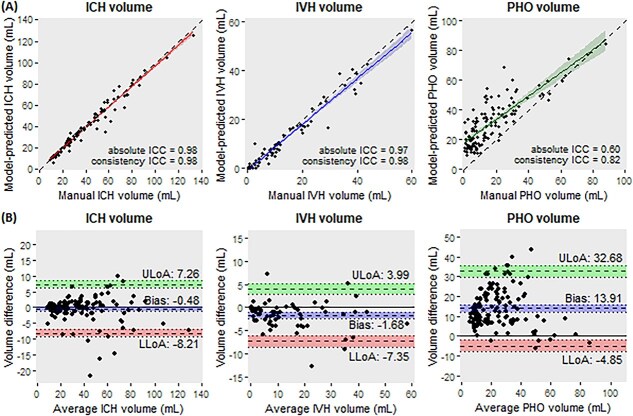
Reliability and agreement between manual and model-predicted segmentations of intracerebral haemorrhage (ICH), intraventricular haemorrhage (IVH) and perihaematomal oedema (PHO). (A) Scatterplots with regression lines and intraclass correlation coefficients (ICCs) for ICH, IVH and PHO. Dashed lines represent the line of identity for reference. (B) Bland–Altman plots showing the bias and the lower (LLoA) and upper limits of agreement (ULoA). Shaded areas indicate 95% confidence intervals for the bias and agreement limits.

Performance metrics for the test set are summarised in Table 2. The model achieved a median DSC for ICH of 0.93 (IQR 0.91–0.94), for IVH of 0.75 (IQR 0.57–0.82) and for PHO of 0.53 (IQR 0.34–0.65). Volume correlations between the model and manual segmentations were strong for ICH (mean absolute and consistency ICC both 0.98 [95% CI, 0.98–0.99]) and IVH (absolute ICC 0.97 [95% CI, 0.92–0.98]; consistency ICC 0.98 [95% CI, 0.96–0.99]), but modest for PHO (absolute ICC 0.60 [95% CI, −0.08 to 0.85]; consistency ICC 0.82 [95% CI, 0.76–0.87]) (Figure 4A). Bland–Altman analyses showed minimal bias for ICH and IVH, whereas PHO volumes were systematically overestimated by the model (bias 13.91 mL [limit of agreement −4.85 to 32.68]) (Figure 4B).

**Table 2:** Performance metrics of the derived model on the test set.

		ICH	IVH	PHO
**DSC** ^a^		0.93 [0.91–0.94]	0.75 [0.57–0.82]	0.53 [0.34–0.65]
**ICC** ^b^	Absolute ICC	0.98 (0.98–0.99)	0.97 (0.92–0.98)	0.60 (−0.08 to 0.85)
	Consistency ICC	0.98 (0.98–0.99)	0.98 (0.96–0.99)	0.82 (0.76–0.87)
**Bland-Altman** ^b^	Bias, mL	−0.48 (−1.14 to 0.19)	−1.68 (−2.39 to −0.98)	13.91 (12.25–15.58)
	LLoA, mL	−8.21 (−9.34 to −7.07)	−7.35 (−8.56 to −6.14)	−4.85 (−7.71 to −2.00)
	ULoA, mL	7.26 (6.12–8.39)	3.99 (2.77–5.20)	32.68 (29.82–35.54)

Abbreviations: DSC = Dice similarity coefficient; ICH = intracerebral haemorrhage; ICC = intraclass correlation coefficient; IVH = intraventricular haemorrhage; LLoA = lower limit of agreement; PHO = perihaematomal oedema; ULoA = upper limit of agreement.

a Results presented as median [IQR].

b Results excluding influential outliers (2 for ICH, 1 for IVH and 8 for PHO), presented as mean (95% CI).

Two outliers for ICH, one for IVH and eight for PHO were identified (Figure S2) and excluded from the primary ICC and Bland–Altman analyses. Results of the analyses including these outliers are provided in Table S1 and Figure S3.

### Subgroup analyses

Subgroup analyses stratified by ICH location and IVH presence showed variation in baseline characteristics as well as segmentation performance.

In the test set, participants with lobar ICH had larger median ICH (38.9 vs. 22.3 mL) and PHO volumes (22.4 vs. 6.5 mL), and more frequent subarachnoid extension (60.9% vs. 3.9%) compared to those with deep ICH (Table S2). In contrast, deep ICH was more often associated with intraventricular extension (61.0% vs. 34.4%) and larger IVH volumes (11.8 vs. 4.1 mL) (Table S2). For ICH segmentation, the model achieved similarly high DSCs in both deep and lobar haemorrhages (median 0.94 [IQR 0.92–0.95] vs. 0.92 [IQR 0.88–0.94]). IVH segmentation performed better in deep ICH (median DSC 0.79 [IQR 0.66–0.84]) compared to lobar ICH (median DSC 0.59 [IQR 0.22–0.70]). Conversely, PHO segmentation was more accurate in lobar ICH (median DSC 0.62 [IQR 0.52–0.72]) than in deep ICH (median DSC 0.39 [IQR 0.29–0.55]).

Participants in the test set with IVH were more likely to have deep ICH (68.1% vs. 41.7%) compared to those without IVH (Table S3). Segmentation performance was similar for ICH regardless of IVH presence (with IVH: median DSC 0.93 [IQR 0.90–0.94]; without IVH: 0.94 [IQR 0.91–0.95]). For PHO, segmentation performance was slightly lower in patients with IVH (median DSC 0.49 [IQR 0.30–0.63]) compared to those without IVH (median DSC 0.55 [IQR 0.35–0.66]).

## Discussion

We developed and externally validated a 3D U-Net model for simultaneous segmentation of ICH, IVH and PHO on NCCT in patients with spontaneous ICH using multicentre, multivendor datasets. The model achieved excellent performance for ICH, with high spatial overlap and strong volumetric agreement with manual segmentations. For IVH, performance was moderate, showing reliable volumetric correlations but limited by small false-positive segmentations and undersegmentation of IVH with lower density. PHO segmentation was less accurate, with systematic overestimation compared to the manual segmentations. Subgroup analyses showed stable ICH performance across haematoma locations and IVH presence, whereas IVH segmentation performed better in deep than lobar haemorrhages, and PHO segmentation was more accurate in lobar than deep ICH.

Multiple previous studies have investigated deep learning approaches for ICH segmentation, but only few addressed simultaneous segmentation of ICH, IVH and PHO.^39^^,^^40^^,^^49^ One study reported a 3D U-Net trained on a single-centre cohort (*n* = 300) and achieved comparable results for ICH (median DSC 0.92), but higher DSCs for IVH (0.79) and PHO (0.71).^40^ However, external validation was limited as test set segmentations were generated by the same raters. Another study described multiple neural networks trained on a large multicentre dataset (*n* = 1558) and found high DSCs for ICH (0.92) and PHO (0.66), and perfect scores for IVH (1.00), though performance was likely overestimated due to an unconventional DSC calculation, while the lack of external validation restricts conclusions about generalisability.^39^ A third study trained a 3D-U Net on a multicentre dataset (*n* = 775) and performed external validation using data from another centre, reporting median DSCs of 0.85 for ICH, 0.62 for IVH and 0.61 for PHO.^49^ However, interpretation of the findings is complicated by some ambiguities in dataset labelling and the reporting of results.

The differences in segmentation performance across ICH, IVH and PHO observed in our study and others can be explained by both imaging and anatomical factors. ICH is generally well-defined on NCCT due to high contrast of blood in the acute phase against surrounding brain tissue, which allows clear delineation of haematoma boundaries and likely explains the consistently high DSCs across studies. Nonetheless, performance may be reduced in more heterogeneous ICH, such as those with irregular margins or hypodense components, or in cases with intraventricular or extra-axial extension, where boundaries are less distinct. These challenges were also evident in our visual assessments, with misclassification at ventricular borders and in haematomas with density heterogeneity. IVH segmentation is more challenging, as IVH can be fragmented by gravitational displacement within the ventricular system and may appear less dense due to mixture with cerebrospinal fluid. PHO segmentation is inherently the most complex, as oedema appears as gradual hypoattenuation surrounding the ICH with often ill-defined margins on NCCT. As a result, PHO segmentation is known to exhibit substantial interrater variability, with previously reported interrater DSCs as low as 0.57 and 0.69.^38^^,^^40^ In our study, manual PHO segmentation was performed based on visual assessment by two different raters for the training/validation and test sets, and a formal interrater analysis was not performed. This may have contributed to the moderate model performance for PHO. Although HU threshold-based methods have been proposed to improve consistency,^50^ their added value remains uncertain due to overlap between oedema and normal or abnormal white matter, as well as variability in HU values between CT scanners,^51^^,^^52^ limiting the applicability of fixed thresholds in multivendor datasets such as ours. Nevertheless, such standardised approaches and others aimed at improving PHO reference standards could potentially improve consistency in future studies.

Our study has several strengths. We used multicentre, multivendor datasets that reflect real-world clinical variability in image acquisition, enhancing the robustness and generalisability of the model. External validation was performed on an independent cohort with segmentations from an independent rater, enabling a robust and independent evaluation of model performance beyond the training data. Additionally, the dataset used for model development differed substantially in patient characteristics from the test set, offering a more stringent test of generalisability. The model was also trained with additional labels for SAH and SDH, which likely improved its ability to distinguish intraparenchymal, intraventricular and extra-axial haemorrhage components. Finally, combining quantitative performance metrics with qualitative visual assessment provided complementary insights into common failure patterns and boundary uncertainties, supporting targeted model refinement in future research.

This study also has limitations. Differences in study objectives and inclusion criteria between the contributing studies resulted in differences in baseline characteristics between the training/validation and test sets. This may limit the generalisability of the model’s performance across the full clinical spectrum of ICH, particularly for small-volume and infratentorial ICH, which were absent in the test set and for which lower segmentation performance have previously been reported.^18^^,^^40^ Although pooling all scans would have reduced heterogeneity, we deliberately used an independent dataset for external validation to assess true external generalisability. Secondly, the model was specifically developed for segmentation of ICH, IVH and PHO in patients with confirmed ICH, and thus was not trained for ICH detection. Thirdly, only baseline NCCTs were analysed; model performance on follow-up imaging has yet to be established. Fourthly, the reference standard was based on manual segmentations, which do not represent a perfect ground truth. Human error and the inherent difficulty of delineating irregular or ambiguous borders may have introduced label noise, though this also mirrors clinical reality, where inter- and intrarater variability is inevitable. Finally, the model was developed using an earlier version of nnU-Net, which may lack optimisations introduced in more recent releases. The 3D U-Net architecture also has inherent limitations: it relies on supervised voxel-wise learning requiring large amounts of annotated data and captures only limited global context due to its patch-based design. Recent foundation models trained on large, diverse datasets show promise in addressing these issues through improved contextual understanding and transfer learning, which could further enhance haemorrhage segmentation and reduce annotation dependency in future work.^53^

## Conclusion

In conclusion, this study demonstrates that the developed model enables accurate and automated volumetric segmentation of ICH in patients with spontaneous supratentorial ICH, while IVH and PHO segmentation remain more challenging. In research settings, these automated segmentations can already serve as reliable pre-segmentations to reduce time and variability in volumetric analyses, provided they are visually checked and corrected if necessary. Further optimisation and validation are needed before adoption in clinical practice. The trained model has been made publicly available for research use on the Grand Challenge platform (https://grand-challenge.org/algorithms/ich-phe-and-ivh-segmentation).

## Supplementary Material

aakag007_Automated_ICH_IVH_and_PHO_segm_ESJ_Supplementary_material

## Data Availability

The data that support the findings of this study are available from the corresponding author on reasonable request. The developed model is publicly available for research use on the Grand Challenge platform (https://grand-challenge.org/algorithms/ich-phe-and-ivh-segmentation).

## Clinical trial registration

FETCH: not applicable. APACHE-AF: ClinicalTrials.gov: NCT02565693. Dutch ICH Surgery Trial (DIST) pilot study: ClinicalTrials.gov: NCT03608423.
