# Supplementary material for: Deep learning-based automated segmentation of intracerebral haemorrhage, intraventricular haemorrhage and perihaematomal oedema on non-contrast CT
Source: Eur Stroke J. 2026 Mar 7;11(3):aakag007. doi: 10.1093/esj/aakag007 (PMC12965810; doi:10.1093/esj/aakag007)
Supplement: aakag007_Automated_ICH_IVH_and_PHO_segm_ESJ_Supplementary_material [file aakag007_automated_ich_ivh_and_pho_segm_esj_supplementary_material.docx]

Supplementary material to:

Deep learning-based automated segmentation of intracerebral haemorrhage, intraventricular haemorrhage, and perihaematomal oedema on non-contrast CT

Floor N.H. Wilting, Jules P.J. Douwes, Ajay Patel, Floris H.B.M. Schreuder, Ruben Dammers, Gerjon Hannink, Wilmar M.T. Jolink, Sjoert A.H. Pegge, Lotte Sondag, Marieke J.H. Wermer, H. Bart van der Worp, Frederick J.A. Meijer, Catharina J.M. Klijn.

**Table of contents**

[1. **3D U-Net architecture** 2](#_Toc210228809)

[Figure S1. 3D U-Net architecture. 2](#_Toc210228810)

[2. **Details concerning model performance evaluation** 3](#_Toc210228811)

[2.1. Dice Similarity Coefficient (DSC) 3](#_Toc210228812)

[2.2. Intra-class correlation (ICC) 3](#_Toc210228813)

[2.3. Bland-Altman analyses 4](#_Toc210228814)

[3. **Identification of influential outliers** 5](#_Toc210228815)

[Figure S2. Identification of influential outliers. 5](#_Toc210228816)

[4. **ICC and Bland-Altman analyses using all data points (including outliers)** 6](#_Toc210228817)

[Table S1. ICC and Bland-Altman analyses using all data points 6](#_Toc210228818)

[Figure S3. Bland-Altman plots using all data points. 6](#_Toc210228819)

[5. **Subgroup analyses: participant characteristics stratified by ICH location and IVH presence** 7](#_Toc210228820)

[Table S2. Participant characteristics stratified by ICH location (deep versus lobar) 7](#_Toc210228821)

[Table S3. Participant characteristics stratified by IVH (with versus without) 7](#_Toc210228822)

[6. **References** 8](#_Toc210228823)

# 1. 3D U-Net architecture


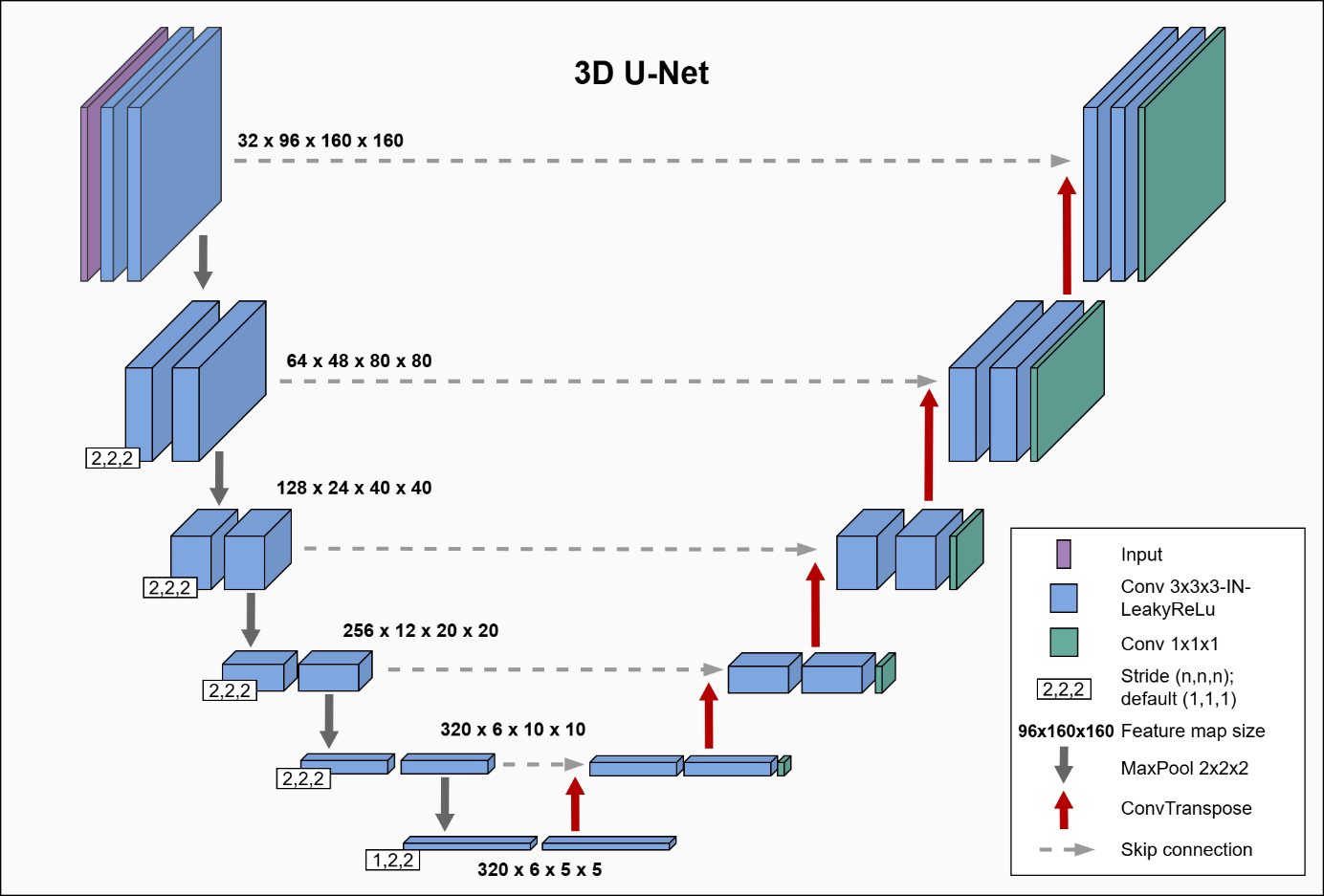


## **Figure S1**. 3D U-Net architecture.

Conv = convolution, IN = Instance Normalization, LeakyReLu = Leaky Rectified Linear Unit, ConvTranspose = transposed convolution.

# 2. Details concerning model performance evaluation

## 2.1. Dice Similarity Coefficient (DSC)

The **Dice Similarity Coefficient (DSC)**, also known as the Sørensen-Dice coefficient, is a *similarity* measure used to quantify the spatial overlap between two segmentations.^1^ The DSC is widely applied in medical image analysis to evaluate segmentation performance by comparing model-predicted segmentations to manual ground truth segmentations.

The DSC is defined as:

$$DSC= \frac{2|A\cap B|}{|A|+|B|}$$

where $\left| A \right|$ denotes the model-predicted segmentation, $\left| B \right|$ the manual ground truth, and $\left| A\cap B \right|$ their intersection. The DSC ranges from 0 (no overlap) to 1 (perfect overlap).

There are no universally accepted cut-off values for interpreting DSC scores, as interpretation depends on several factors, including anatomical complexity of the region of interest, image quality and inter-observer variability.^2^ DSC values should therefore be interpreted within the specific clinical and anatomical context, and ideally compared to human interrater variability.

## 2.2. Intra-class correlation (ICC)

The **intraclass correlation coefficient (ICC)** is a *reliability* metric that quantifies the degree to which two measurement methods produce consistent results across patients.^3^ In the context of image segmentation, ICC is used to assess whether the model and manual rater assign similar volumes and to what extent these volumes are correlated.

Reliability is defined as the proportion of total variance attributable to true between-patient differences rather than measurement error^4^:

$$reliability=\frac{betweenpatient variance}{betweenpatient variance+measurement error}=\frac{\sigma_{p}^{2}}{\sigma_{p}^{2}{+\sigma}_{m}^{2}+\sigma_{e}^{2}}$$

where $\sigma_{p}^{2}$ denotes between-patient variance, $\sigma_{m}^{2}$ variance due to systematic differences between the model and the manual rater, and $\sigma_{e}^{2}$ variance due to random error.

Depending on how measurement error is defined, two types of ICC are distinguished^3-5^:

- **Absolute ICC**: accounts for both systematic and random error, and reflects the extent to which the model and manual rater assign similar volumes.

$${ICC}_{absolute}=\frac{\sigma_{p}^{2}}{\sigma_{p}^{2}{+\sigma}_{m}^{2}+\sigma_{e}^{2}}$$

- **Consistency ICC**: accounts only for random error, and reflects the correlation between the volumes assigned to patients by the model and the manual rater.

$${ICC}_{consistency}=\frac{\sigma_{p}^{2}}{\sigma_{p}^{2}+\sigma_{e}^{2}}$$

ICC values range from 0 to 1, with higher values indication greater reliability. There are no standard cut-off values for acceptable reliability using ICC, but suggested interpretive thresholds are:^5^

| ICC value | Interpretation |
| --- | --- |
| < 0.50 | poor reliability |
| 0.50 – 0.74 | moderate reliability |
| 0.75 – 0.89 | good reliability |
| ≥ 0.90 | excellent reliability |

## 2.3. Bland-Altman analyses

The **Bland-Altman analysis** is a graphical method used to assess *agreement* between two quantitative measurement methods.^6^ For model evaluation, it is used to evaluate volumetric agreement between model-predicted and manually segmented volumes.

In a Bland-Altman plot, the difference in volume between the model-predicted and manual segmentations is plotted against the mean. The mean volume difference, referred to as the bias, is calculated, along with the corresponding 95% limits of agreements (LoA), defined as:

$$LoA=bias\pm1.96s$$

where $s$ denotes the standard deviation of the bias. This approach enables visual inspection of systematic bias and variability across the measurement range.

# 3. Identification of influential outliers

**DFBETAS** (short for Difference in Betas) are diagnostic measures in regression analysis used to identify influential data points: observations that have a large effect on the estimated regression coefficients. The DFBETA measures the influence of a single data point on the coefficient estimate ($\beta$) by removing each data point and recomputing the coefficients. A high DFBETA suggests that the corresponding data point may be an outlier or leverage point, potentially distorting the results or conclusions.

A data point was classified as influential if its DFBETA for the intercept exceeded the threshold $\left| \mathrm{DFBETA} \right|>2/\sqrt{n}$, where $n$ is the sample size.^7^


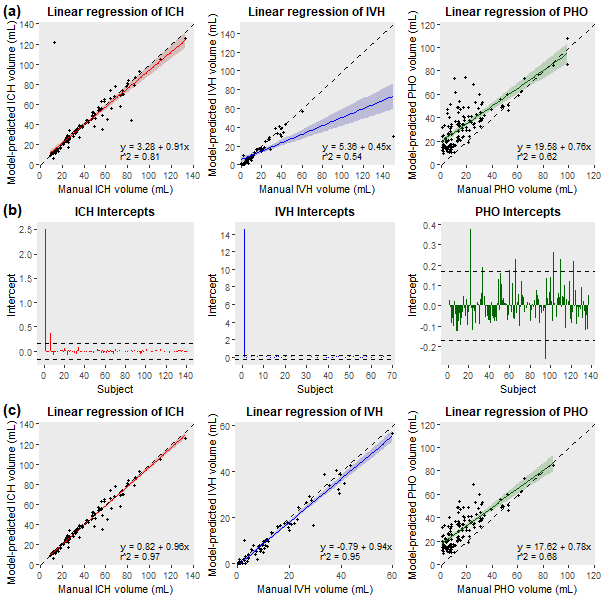


## **Figure S2.** Identification of influential outliers.

**(a)** Linear regression models of intracerebral haemorrhage (ICH), intraventricular haemorrhage (IVH) and perihaematomal oedema (PHO) including all measurements. **(b)** DFBETA values illustrating the influence of individual measurements on the estimated y-intercepts for ICH, IVH and PHO. Horizontal references lines indicate the threshold for influential observations (±2/$\surd n$, with $n$ representing the sample size). **(c)** Linear regression models after exclusion of influential outliers: two for ICH, one for IVH and eight for PHO.

# 4. ICC and Bland-Altman analyses using all data points (including outliers)

## **Tab****le S1**. ICC and Bland-Altman analyses using all data points

|  | | ICH | IVH | PHO |
| --- | --- | --- | --- | --- |
| ICC | Absolute ICC | 0.90 (0.86 to 0.93) | 0.65 (0.49 to 0.77) | 0.59 (-0.05 to 0.83) |
|  | Consistency ICC | 0.90 (0.86 to 0.93) | 0.65 (0.50 to 0.77) | 0.79 (0.71 to 0.84) |
| Bland-Altman | Bias, mL | 0.03 (-1.73 to 1.78) | -3.42 (-6.96 to 0.12) | 15.23 (13.17 to 17.29) |
|  | LLoA, mL | -20.64 (-23.65 to -17.64) | -32.10 (-38.18 to -26.01) | -8.65 (-12.18 to -5.12) |
|  | ULoA, mL | 20.69 (17.69 to 23.70) | 25.26 (19.17 to 31.34) | 39.11 (35.58 to 42.63) |
| ICH: intracerebral haemorrhage; ICC: intraclass correlation coefficient; IVH: intraventricular haemorrhage; LLoA: lower limit of agreement; PHO: perihaematomal oedema; ULoA: upper limit of agreement.  All results presented as mean (95% CI). | | | | |


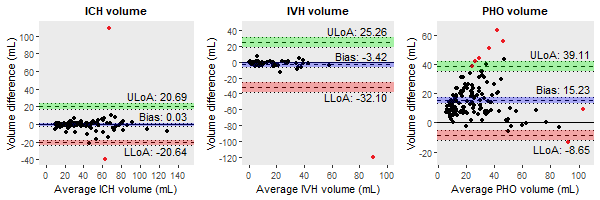


## **Figure S3.** Bland-Altman plots using all data points.

Bland-Altman plots showing the bias and lower (LLoA) and upper limits of agreement (ULoA) between manual and model-predicted segmentations of intracerebral haemorrhage (ICH), intraventricular haemorrhage (IVH) and perihaematomal oedema (PHO). Influential outliers are highlighted in red. Shaded areas present 95% confidence intervals for the bias and agreement limits.

# 5. Subgroup analyses: participant characteristics stratified by ICH location and IVH presence

## **Table S2**. Participant characteristics stratified by ICH location (deep versus lobar)

| Characteristic | Training/validation set ($\boldsymbol{n}$ = 301)^*^ | | Test set ($\boldsymbol{n}$ = 141) | |
| --- | --- | --- | --- | --- |
|  | Deep ICH ($n$=132) | Lobar ICH ($n$=113) | Deep ICH ($n$=77) | Lobar ICH ($n$=64) |
| Age, years; median [IQR] | 70 [56 to 79] | 72 [64 to 79] | 63 [54 to 70] | 72 [63 to 81] |
| Sex; $\boldsymbol{n}$ (%) |  |  |  |  |
| Male | 86 (65.2) | 73 (64.6) | 54 (70.1) | 35 (54.7) |
| Female | 46 (43.8) | 40 (35.4) | 23 (29.9) | 29 (45.3) |
| Intraventricular extension; $\boldsymbol{n}$ (%) | 48 (36.4) | 44 (38.9) | 47 (61.0) | 22 (34.4) |
| Subarachnoid extension; $\boldsymbol{n}$ (%) | 25 (18.9) | 42 (37.2) | 3 (3.9) | 39 (60.9) |
| Subdural extension; $\boldsymbol{n}$ (%) | 5 (3.8) | 3 (2.7) | 0 (0) | 3 (4.7) |
| ICH volume, mL; median [IQR] | 6.6 [3.0 to 18.3] | 12.3 [4.6 to 33.6] | 22.3 [12.6 to 35.0] | 38.9 [26.5 to 57.4] |
| IVH volume, mL; median [IQR] | 3.7 [1.5 to 21.3] | 3.3 [1.2 to 9.0] | 11.8 [5.8 to 25.0] | 4.1 [1.5 to 9.5] |
| PHO volume, mL; median [IQR] | 9.7 [6.0 to 20.5] | 13.0 [6.6 to 33.5] | 6.5 [4.1 to 12.5] | 22.4 [13.7 to 31.6] |
| ICH: intracerebral haemorrhage; IVH: intraventricular haemorrhage; IQR: interquartile range; n: number; PHO: perihaematomal oedema.  ^*^ one patient had a primary intraventricular haemorrhage without intraparenchymal involvement and 55 patients had an infratentorial ICH; these patients are therefore not included in this table. | | | | |

## **Table S3**. Participant characteristics stratified by IVH (with versus without)

| Characteristic | Training/validation set ($\boldsymbol{n}$ = 301) | | Test set ($\boldsymbol{n}$ = 141) | |
| --- | --- | --- | --- | --- |
|  | IVH ($n$=121) | No IVH ($n$=180) | IVH ($n$=69) | No IVH ($n$=72) |
| Age, years; median [IQR] | 70.5 [61 to 77.3] | 73 [62 to 80] | 65 [57 to 76] | 66.5 [59 to 75.5] |
| Sex; $\boldsymbol{n}$ (%) |  |  |  |  |
| Male | 82 (67.8) | 110 (61.1) | 42 (60.9) | 47 (65.3) |
| Female | 39 (32.2) | 70 (38.9) | 27 (39.1) | 25 (34.7) |
| ICH location; $\boldsymbol{n}$ (%) |  |  |  |  |
| Lobar | 44 (36.4) | 69 (38.3) | 22 (31.9) | 42 (58.3) |
| Deep | 48 (39.7) | 84 (46.7) | 47 (68.1) | 30 (41.7) |
| Infratentorial | 29 (23.1) | 27 (15.0) | 0 (0) | 0 (0) |
| Subarachnoid extension; $\boldsymbol{n}$ (%) | 31 (25.6) | 50 (27.8) | 16 (23.2) | 26 (36.1) |
| Subdural extension; $\boldsymbol{n}$ (%) | 5 (4.1) | 4 (2.2) | 1 (1.4) | 2 (2.8) |
| ICH volume, mL; median [IQR] | 10.0 [2.3 to 28.3] | 7.6 [3.5 to 19.1] | 30.6 [15.7 to 50.4] | 26.5 [16.7 to 43.6] |
| IVH volume, mL; median [IQR] | 3.1 [1.2 to 12.4] | n.a. | 9.7 [3.9 to 20.9] | n.a. |
| PHO volume, mL; median [IQR] | 11.0 [6.7 to 27.5] | 11.1 [5.7 to 21.9] | 10.5 [5.1 to 26.4] | 13.8 [6.4 to 22.3] |
| ICH: intracerebral haemorrhage; IVH: intraventricular haemorrhage; IQR: interquartile range; n: number; n.a.: not applicable; PHO: perihaematomal oedema. | | | | |

# 6. References

1. Dice LR. Measures of the amount of ecologic association between species. *Ecology*. 1945;26(3):297-302. doi:10.2307/1932409

2. Kocak B, Klontzas ME, Stanzione A, et al. Evaluation metrics in medical imaging AI: fundamentals, pitfalls, misapplications, and recommendations. *Eur J Radiol Artif Intell*. 2025;3:100030. doi:10.1016/j.ejrai.2025.100030

3. Haghayegh S, Kang HA, Khoshnevis S, Smolensky MH, Diller KR. A comprehensive guideline for Bland-Altman and intra class correlation calculations to properly compare two methods of measurement and interpret findings. *Physiol Meas*. Jun 15 2020;41(5):055012. doi:10.1088/1361-6579/ab86d6

4. Liljequist D, Elfving B, Skavberg Roaldsen K. Intraclass correlation - A discussion and demonstration of basic features. *PLoS One*. 2019;14(7):e0219854. doi:10.1371/journal.pone.0219854

5. Koo TK, Li MY. A Guideline of Selecting and Reporting Intraclass Correlation Coefficients for Reliability Research. *J Chiropr Med*. Jun 2016;15(2):155-63. doi:10.1016/j.jcm.2016.02.012

6. Giavarina D. Understanding Bland Altman analysis. *Biochem Med (Zagreb)*. 2015;25(2):141-51. doi:10.11613/BM.2015.015

7. Cohen J, Cohen P, West SG, Aiken SL. *Applied multiple regression/correlation analysis for the behavioral sciences*. Third ed. Lawrence Erlbaum Associates; 2003.
